# Bone histology sheds new light on the ecology of the dodo (*Raphus cucullatus*, Aves, Columbiformes)

**DOI:** 10.1038/s41598-017-08536-3

**Published:** 2017-08-24

**Authors:** D. Angst, A. Chinsamy, L. Steel, J. P. Hume

**Affiliations:** 1 Department of Biological Sciences, University of Cape Town, Private Bag X3, Rhodes Gift, 7701 South Africa; 2 Department of Earth Sciences, Natural History Museum, Cromwell Road, London, SW7 5BD United Kingdom; 3 Bird Group, Department of Life Sciences, Natural History Museum, Akeman Street, Tring, Herts HP23 6AP United Kingdom

## Abstract

The dodo, *Raphus cucullatus*, a flightless pigeon endemic to Mauritius, became extinct during the 17^th^ century due to anthropogenic activities. Although it was contemporaneous with humans for almost a century, little was recorded about its ecology. Here we present new aspects of the life history of the dodo based on our analysis of its bone histology. We propose that the dodo bred around August and that the rapid growth of the chicks enabled them to reach a robust size before the austral summer or cyclone season. Histological evidence of molting suggests that after summer had passed, molt began in the adults that had just bred; the timing of molt derived from bone histology is also corroborated by historical descriptions of the dodo by mariners. This research represents the only bone histology analysis of the dodo and provides an unprecedented insight into the life history of this iconic bird.

## Introduction

The dodo (*Raphus cucullatus*), a giant flightless pigeon, was endemic to Mauritius^1^, one of the Mascarene Islands in the southwestern Indian Ocean. It was first described in 1598 by Dutch mariners^2^, and became extinct less than a hundred years later (between 1662 and 1693)^3, 4^. It has now become an icon of extinction and a classic symbol of a human-induced extinction event. Although over hunting has been cited as a cause of the dodo’s demise, the introduction of invasive mammals, especially monkeys, deer, pigs and rats were primarily responsible^3^.

The skeletal anatomy of the dodo has been well studied^5^, and more recently Claessens *et al*.^6^ used novel methods to further understand its anatomy. Taxonomic studies have identified the dodo as a member of Columbiformes, and as the sister taxon to the Solitaire (*Pezophaps solitaria*)^1, 7^, but the ecology of the dodo has remained difficult to determine. The latter is perhaps explained by the fact that living Columbiformes differ considerably from the dodo making ecological comparisons difficult, and perhaps more significantly, the dodo became extinct long before any detailed ecological studies were carried out on Mauritius. Although some direct observations of the dodo are available from the 16^th^ and 17^th^ century, these are by sailors with no scientific background and are mainly contradictory and imprecise^8^. A number of contemporary illustrations of the dodo are known, but most are considered unreliable^8^. Recent body mass estimates range from 9.5 kg to 14.3 kg^9–12^; but almost no other information exists. Our research is therefore highly significant in that using bone microstructure we provide novel insight into the reproductive behavior, growth strategy, and molting habits of this recently extinct enigmatic bird^13^.

## Results

The histological structure of the bones from the Mare aux Songes (MAS) locality is generally much better preserved than subfossils recovered from the cave environments (Supplementary Table S1).

### Ontogenetic trajectory

**Figure 1:**
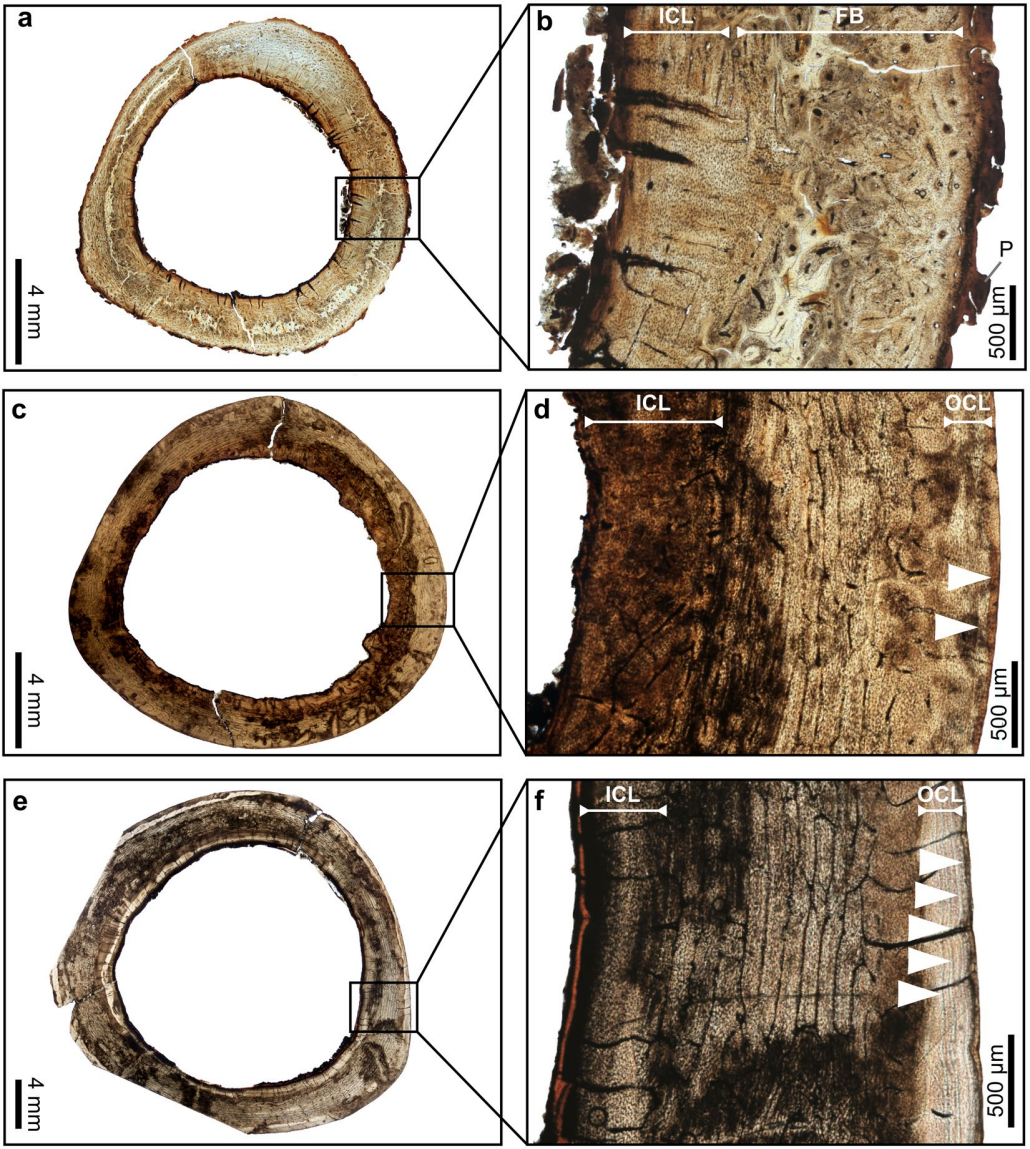
Transverse thin sections of hindlimb bones of the dodo showing different stages of the ontogenetic growth series. (**a**) Overview of a thin section of the tibiotarsus of a juvenile (ddtbt14). (**b**) Detail of the framed region in (a). (﻿**c**) Overview of a thin section of the tibiotarsus of young adult with 2 LAGs (ddtbt13). (**d**) Detail of the framed region in (c). (**e**) Overview of a thin section of the tibiotarsus of a more mature adult with 5 LAGs (ddtbt10). (**f**) Detail of the framed region in (e). White arrows: Lines of arrested growth, P: remnants of the periosteum, FB: Fibrolamellar bone, ICL: Inner Circumferential Layer, OCL: Outer Circumferential Layer.

The histology of the dodo is similar to that of modern birds, which generally have three distinct layers that make up the bone wall^12, 13^. The central layer typically consists of fibrolamellar bone, which is a rapidly-deposited woven tissue, rich in primary osteons. This is overlain by a poorly vascularized or avascular tissue termed an outer circumferential layer (OCL), and underlain by an inner circumferential layer (ICL), which is composed of more slowly deposited lamellar bone tissue (terminology sensu Chinsamy-Turan^13^; Fig. 1c–f, Supplementary Table S2).

Our sample of dodo bones shows different stages of growth and maturity. Tibiotarsus (ddtbt14), has a cortex largely comprising of fibrolamellar bone tissue consisting of woven bone with many primary osteons, which suggests a rapid rate of bone deposition. Remnants of the periosteum along the outer surface are preserved and an inner circumferential layer (ICL), without resorption traces, is well developed (Fig. 1a,b, Supplementary Table S2). These characteristics indicate a late juvenile stage that has passed the most rapid phase of growth, but one that has not yet reached adulthood^13, 14^, making it the youngest specimen in our sample; all the other specimens studied appear to be adults, at slightly different stages of maturity.

**Figure 2:**
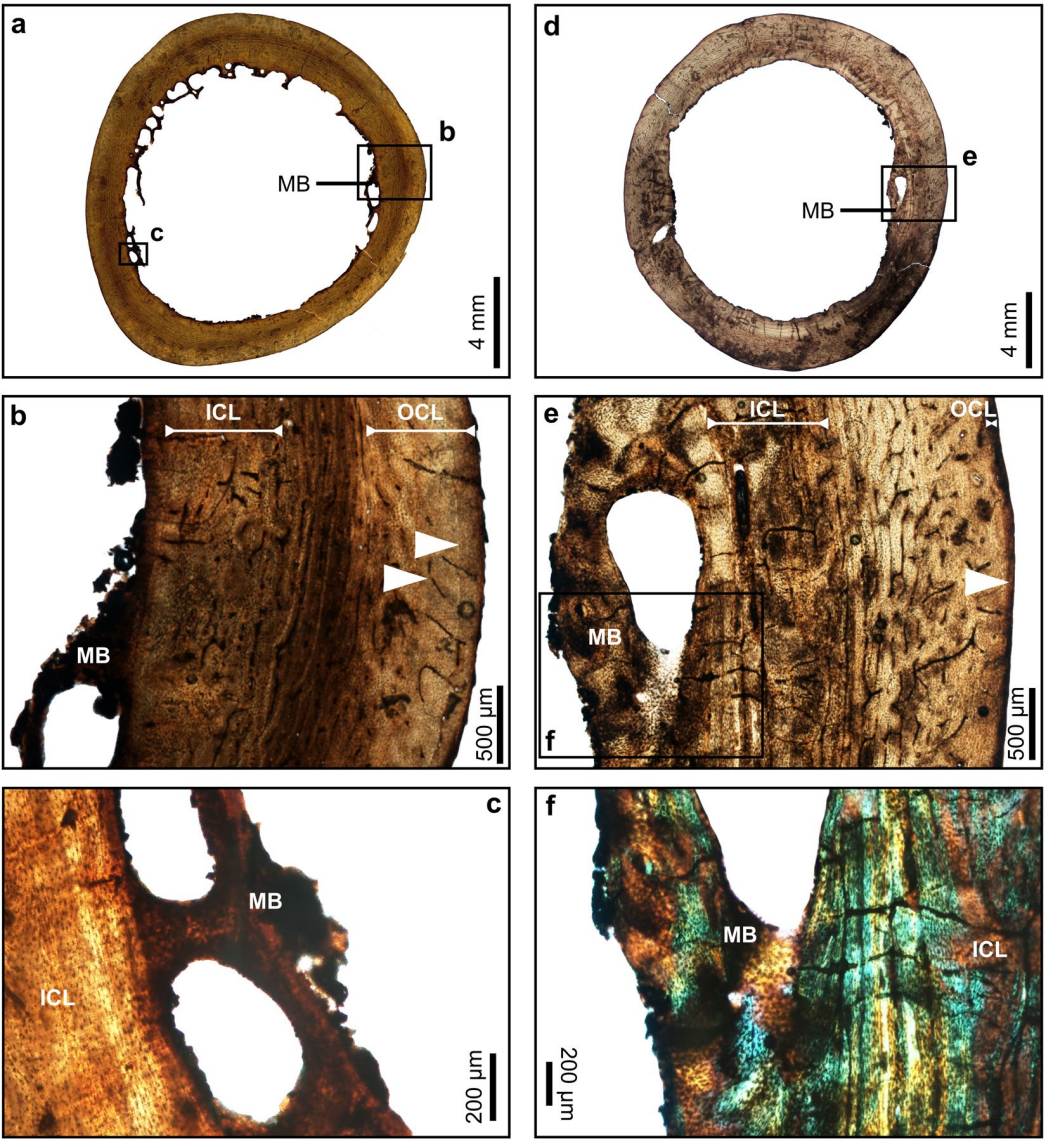
Transverse thin sections of hindlimb bones of the dodo showing medullary bone. (**a**) Overview of a thin section of the tibiotarsus showing medullary bone extending from the ICL (ddtbt08). (**b**) Detail of the framed region in (a). (**c**) Detail of the framed region in (a) under crossed-polarized light﻿, showing that the resorption is in the medullary bone, which has a distinctive woven bone structure, different to the lamellar bone observed for the ICL. (**d**) Overview of a thin section of a femur of a young adult with 1 LAG in the OCL and medullary bone extending from the ICL (ddfem04). (**e**) Detail of the framed region in (**d**). (**f**) Detail of the framed region in (d) under crossed-polarized light, showing that the resorption is in the medullary bone, which consists of woven bone. White arrows: Lines of arrested growth, ICL: Inner Circumferential Layer, OCL: Outer Circumferential Layer, MB: Medullary Bone.

**Figure 3:**
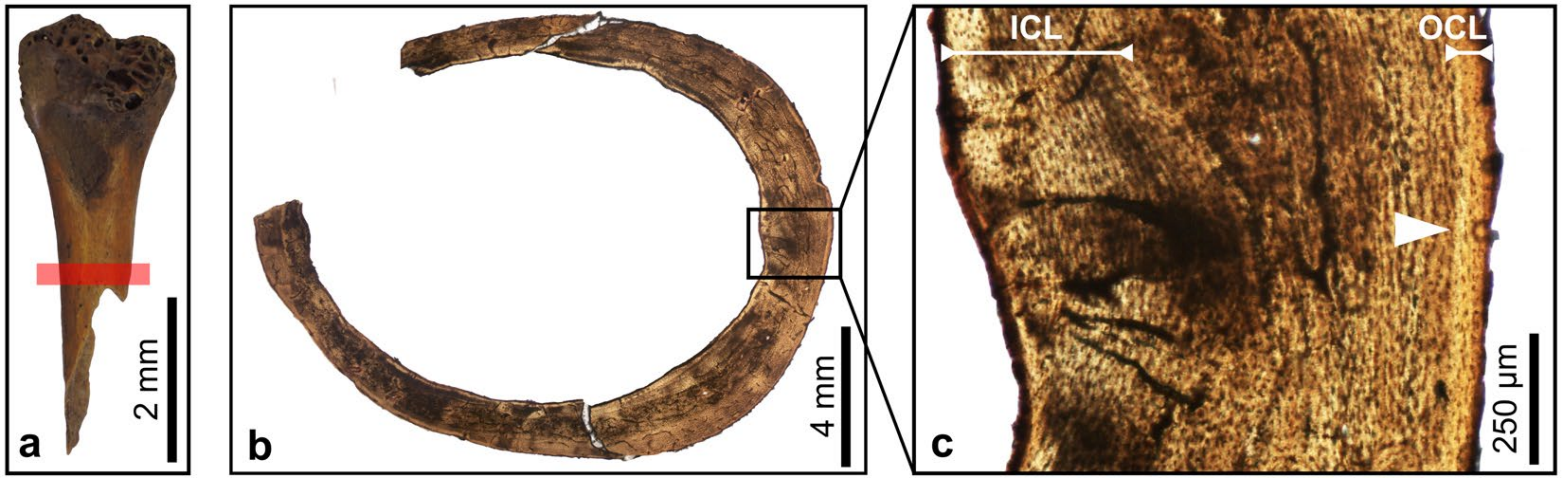
Transverse thin sections of a humerus of the dodo. (**a**) Specimen ddhu01 indicating the location of the sectioning (red bar). (**b**) Overview of a thin section of the humerus (ddhu01). (**c**) Detail of the framed region in (b). White arrow: Line of arrested growth, ICL: Inner Circumferential Layer, OCL: Outer Circumferential Layer.

**Figure 4:**
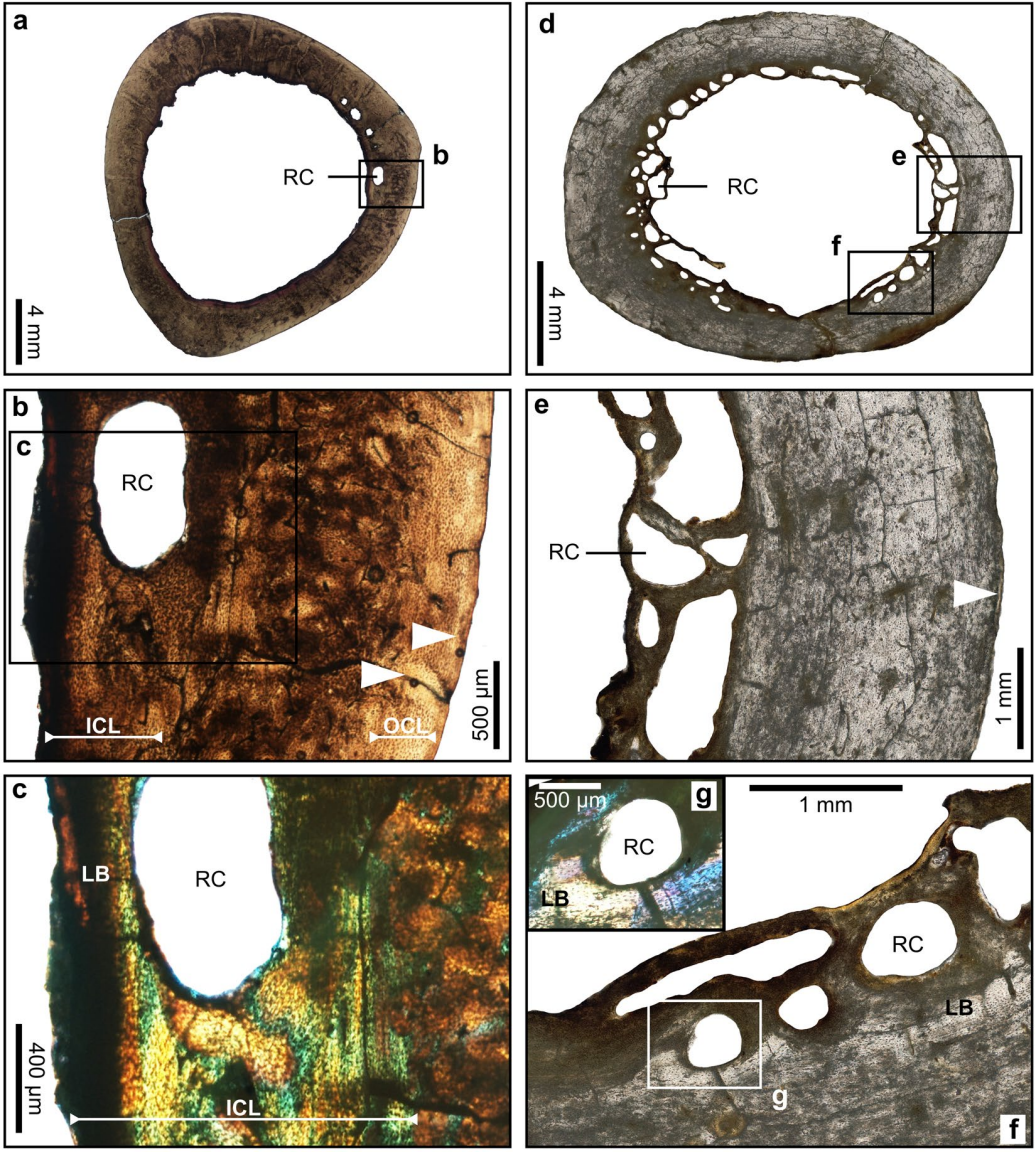
Transverse sections of hindlimb bones of the dodo showing resorption cavities which are interpreted as evidence of molt. (**a**) Overview of a tibiotarsus of a mature adult with enlarged cavities (ddtbt06). (**b**) Detail of the framed region in (a). (**c**) Detail of the framed region in (a) under crossed-polarized light, showing that the resorption cavities are in the lamellar bone tissue of the ICL. (**d**) Overview of a thin section of a femur showing a large amount of resorption cavities (ddfem03). (**e**) Detail of the framed region in (**d**). (**f)** Detail of the framed region in (**d**). (**g**) Detail of the framed region in (f) under crossed-polarized light, showing that the resorption cavities are in the lamellar bone tissue of the ICL.White arrows: Lines of arrested growth, ICL: Inner Circumferential Layer, OCL: Outer Circumferential Layer, RC: Resorption Cavity, LB: Lamellar Bone.

**Figure 5:**
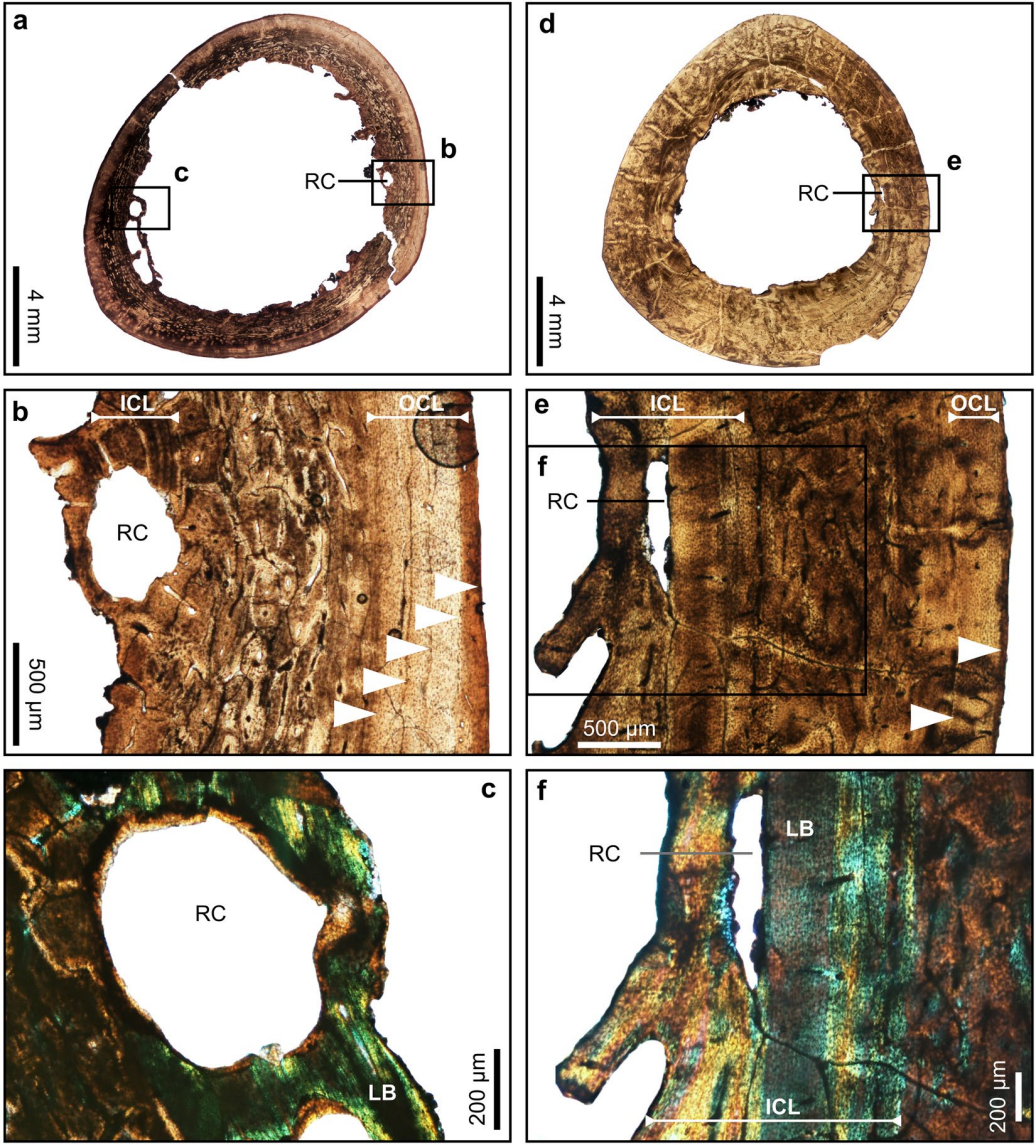
Transverse sections of hindlimb bones of the dodo showing resorption cavities which are interpreted as evidence of molt. (**a**) Overview of a thin section of a tibiotarsus of a mature adult (ddtbt2). The endosteal margin is clearly resorptive, and several resorption cavities are evident. (**b**) Detail of the framed region in (a). (**c**) Detail of the framed region in (a) under crossed-polarized light, showing that the resorption cavities are mainly in the lamellar bone tissue of the ICL, and extend into the fibrolamellar bone. (**d**) Overview of a thin section of the tibiotarsus of a young adult showing a resorptive endosteal margin, and resorption cavities (ddtbt5). (**e**) Detail of the framed region in (d). (**f**) Detail of the framed region in (e) under crossed-polarized light, showing that the resorption cavities are located in the lamellar bone tissue of the ICL. White arrow: Lines of arrested growth, ICL: Inner Circumferential Layer, OCL: Outer Circumferential Layer, LB: Lamellar bone, RC: Resorption Cavity.

Several specimens appear to be young adults in their first year post sexual maturity (ddfem02, ddfem03, ddfem04, ddfem05, ddhu01, ddtbt04). These individuals have a single line of arrested of growth (LAG) and they show the beginning of the development of the OCL [representing about 5% to 20% of the bone wall thickness (BWT; Figs 2d,e, 3 and 4d,e; Supplementary Table S2)], which indicates their skeleton has reached maturity^15–19^. These specimens all show some evidence of endosteal resorption, and they have a well-developed ICL, which comprises 15% to 35% of the cortex thickness (Supplementary Table S2). The rest of the sample (ddfem01, ddtbt01, ddtbt02, ddtbt03, ddtbt05, ddtbt06, ddtbt08, ddtbt09, ddtbt10, ddtbt13) represent more mature adults. They have a well-developed OCL of variable thickness, between 7% to 29% of the BWT (Supplementary Table S2), with two to five/six LAGs (Figs 1c–f, 2a,b, 4a,b and 5). The ICL in these specimens is generally thick, making up 14% to 51% of the BWT (Supplementary Table S2), and is often affected by endosteal resorption (Figs 1c–f, 2a,b, 4 and 5).

Only the tarsometatarsi (ddtmt01 and ddtmt02) show a large amount of secondary reconstruction, which obscures the triple-layer structure of the bone wall. This could be linked to biomechanical adaptations^13^, or possibly suggests the importance of these bones as calcium reservoirs^20^.

### Identification of females

For two of the specimens, one tibiotarsus (ddtbt08) and one femur (ddfem04), a distinctive woven bone tissue (medullary bone)^13^, extends centripetally from the ICL into the medullary cavity (Fig. 2; Supplementary Table S3). This bone tissue develops in ovulating birds and is utilized in the formation of the calcium rich eggshells^3, 19^. The tibiotarsus (ddtbt08) has a large amount of medullary bone, making up about 4.5% of the BWT (Supplementary Table S3), while the femur (ddfem4) preserves medullary bone only in some areas of the bone wall, being approximately 1.5% of the BWT (Supplementary Table S3).

### Osteohistological indicator of molting

Most adult specimens have many secondary osteons or resorption cavities in the compacta. One femur (ddfem03) and four tibiotarsi (ddtbt02, ddtbt05, ddtbt06, ddtbt09) exhibit large erosion cavities in the endosteal part of the cortex (Figs 4 and 5). The resorption cavities have an area of between 0.01 mm² and 0.98 mm², which corresponds to 0.1% to 5.9% of the total bone area (Supplementary Table S3). In some of these enlarged cavities, a centripetal deposition of lamellar bone occurs (Fig. 4b,c).

## Discussion

The youngest individuals in our sample appear to be late stage juveniles. A large proportion of their compacta comprises fibrolamellar bone, which suggests that during early ontogeny bone deposition occurred at a rapid rate^13, 21^. Such rapid rates of growth during the early growth stages is typical for modern birds; it has also been described in other species such as the secretary bird (*Sagittarius serpentarius*)^14^, Japanese quail *(Coturnix japonica)* ^22^, king penguin (*Aptenodytes patagonicus*)^23^ and ostrich (*Struthio camelus*)^14^, as well as in Mesozoic ornithurine birds^13, 24, 25^. The well-developed ICL in the youngest dodo specimens suggests that medullary expansion had already been completed in these individuals^13^.

During later stages of ontogeny, the fibrolamellar bone, which formed during early ontogeny, is reconstructed and remodeled^13, 14^. The compacta of adult individuals show a well-developed ICL and OCL, both consisting of more slowly formed lamellar bone tissue. The formation of the OCL tends to occur after the development of the ICL, and only once sexual maturity has been attained^14, 22^. This three-layer arrangement of the tissue in the bone wall in the dodo is similar to that described by Enlow and Brown^26^ for a modern hawk (*Buteo*), chicken (*Gallus*), turkey (*Meleagris*), guinea fowl (*Numida*) and crow (*Corvus*), and has been shown for other birds such as secretary bird *S. serpentarius* ^14^ and albatross *Diomedea* ^13^.

Like large modern flightless birds, e.g. cassowary (*Casuarius*)^27^, ostrich (*Struthio*)^14^ and rhea (*Rhea*)^28^, the dodo also has rapidly formed fibrolamellar bone tissue. However, unlike these birds in which this is the predominant tissue of the bone wall, in the dodo this tissue makes up only one third of it. Slower, more protracted rates of growth during ontogeny have been documented for moas (Dinornithiformes)^27^, the kiwi (*Apteryx*)^29^, and the Late Cretaceous *Gargantuavis* ^30^. The slower growth to adult size appears to be related to the lack of predators and to environmental resource stress^22, 27, 29, 30^. Interestingly, although the dodo does not exhibit the slow protracted growth of these large island birds, it does appear to have had an extended growth period when reaching skeletal maturity, which is confirmed by the presence of several LAGs in the OCL. Thus, the dodo experienced rapid growth rates until the attainment of sexual maturity, but thereafter it took several years to attain skeletal maturity. Such an extended, slow growth after sexual maturity might have been possible on a small island like Mauritius^24^ where until the arrival of humans, adult birds lacked any natural predators.

Among modern birds, when lines of arrested of growth are present, they tend to be restricted to the OCL^13^. However, terrestrial birds on islands, like the kiwi^29^ or Dinornithiformes^27^, have several LAGs throughout the cortex. Turvey *et al*.^27^ proposed that the LAGs in the slow growing Dinornithiformes, and in particular *Megalapteryx didinus* are the result of severe environmental fluctuations^27^. Likewise, Köhler *et al*.^31^ suggested that the frequent observation of LAGs in wild ruminants were the result of seasonality. In addition, the growth lines evident in the insular *Myotragus* is considered to be the result of resource limitation^32^. Experimental studies on birds that were given restricted access to food have demonstrated that although such limitations did not necessarily produce LAGs, they resulted in a decrease in bone depositional rates^22^. However, if access to food was more severely limited, LAGs may have resulted; this has been suggested for another insular flightless columbiform, the solitaire *Pezophaps solitaria* ^32^. In the case of the dodo, harsh seasonal conditions on Mauritius exist during the summer months, between November and March. During this period, cyclones can occur, during which heavy rain and strong winds can strip trees of leaves, flowers and fruit, causing severe food shortages and starvation for the island fauna, and it can take a few months for normal environmental conditions to return^3, 33, 34^. These events may explain the variations in body mass, as reported by mariners for the dodo^8^. As these seasonal events and consequent long food shortages could result in nutritional stress, we suggest that the LAGs observed in the OCL in the dodo bones were produced in the summer months, between November and March.

The presence of medullary bone in two specimens proves unequivocally that part of the sample were ovulating females^35^. The tibiotarsus (ddtbt08) shows a large area of medullary bone in comparison to the femur (ddfem04). This could be a result of histological variability between these skeletal elements^13^, although they could also suggest differences in their reproductive cycles, *i.e*. one bird (represented by ddtbt08) had not yet utilized the medullary bone for eggshell production, while the other bird (represented by ddfem04) had already shelled its eggs^13, 21, 36^. Livezey^37^ had previously used the K-means statistical method to deduce sexual dimorphism in the dodo; however he did not statistically check the validity of the clusters obtained using the K-means methodology, which therefore invalidates them. Apart from this work, the dodo is not considered to be overly sexually dimorphic, and in the current study we found that the circumferences of the bones of the females were similar to other adult bones in the sample (Supplementary Tables S2–S3). Thus, as for the extinct birds *Confuciusornis* ^35^ and *Dromornis* ^38^, bone histology has proved to be an important tool in sex determination of the dodo.

The adult dodo specimens generally show a large amount of secondary reconstruction, with many secondary osteons present in the cortex, as well as evidence of extensive resorption around the medullary cavity^13^. However, five specimens (ddfem03, ddtbt02, ddtbt05, ddtbt06, ddtbt09), have much enlarged cavities in the cortex suggesting that the demand for calcium surpassed the normal requirements. Such significant bone remobilization has been reported in penguins during molting when the demand for calcium is increased^39, 40^, as well as, for 15 species of modern birds including the domestic pigeon^40^. Periodic molting is common among modern birds^34, 41, 42^, which allows time for the replacement of old damaged feathers, and commonly occurs after breeding^3, 33, 34, 41, 42^. Molting periods have been previously proposed for the dodo^43–47^, but these are generally unsupported^48^. On the basis of earlier osteohistological observations made on several other birds species^39, 40^, we suggest that the presence of extensive resorption in the bones of the dodo could be interpreted as evidence of molting.

We further propose that since molt can generate significant changes in the appearance of birds in terms of color and feather type^3^, this may explain the many discrepancies in the descriptions of the dodo in historical accounts^8, 49^. The dodo was variably described as having “three or four black quills” in the place of their wings, and a tail with “four or five small curled plumes of a greyish color”^49^. Some other descriptions of the dodo mention a “clothing of downy feathers” or even “no feathers on their body, which is covered in black down”^49^. Thus, we propose that mariners may have been describing the dodo at different stages of molt. Dodos described as having a downy plumage^49^ were probably observed and described just after molt, as seen in modern birds in a similar condition^3, 34, 41, 42^, whilst the grey or black plumage^49^ could correspond to dodo specimens between two molting periods^50^.

Our study of bone microstructure has given insight into the molting and reproductive behavior of the dodo, but the timing of these events needs further clarification. Since Mauritius has a seasonal cycle of cyclonic events, which occurs between November and March (austral summer), Staub^44^ suggested that the dodo bred in the austral winter, between March and September, when food was abundant, with a post-breeding molting period between September and December. He based this hypothesis on a description by Captain van Westzanen in 1602, who described the meat of the dodo as a very tasty food. Staub^44^ considered that this culinary aspect was a result of the birds eating a profusion of fruits from the endemic palm trees (*Latania* sp., *Dictyosperma* sp., *Hyophorbe* sp.), which produced fruits during the winter. Thus, he suggested, that the dodo evolved its reproduction period to coincide with food abundance, following the same reproductive strategy of the solitaire on neighboring Rodrigues Island. However, if we compare this annual molting pattern proposed by Staub^44^ with information known about modern surviving birds on Mauritius, his hypothesis is improbable. On Mauritius, (*contra* Staub), all birds breed during the austral summer (between August and January, which sometimes extends into March), and start the post-breeding molt between November and March^3, 33, 34, 41^. Moreover, the Pink Pigeon (*Nesoenas mayeri*), which has the longest molting period of any bird on Mauritius, does not molt during the months towards the end of the austral winter (June to October) when food resources are limited^33^.

Thus, bone histology provides significant information about the timing of breeding and molting periods in the dodo. We can now consider that as LAGs form during the cyclone period between November and March, the breeding and the molting periods can be accurately determined using the amount of bone deposited after the LAG (Fig. 6.1). Assuming that the bone deposition rate is constant between consecutive LAGs, we propose that the thicker the bone deposition between two LAGs, the more time has lapsed since the LAG formed, thereby giving an indication of when the breeding or molting event occurred. Furthermore, if we examine the external cortex of the two specimens that have formed medullary bone (ddtbt08 and ddfem04), it is evident that a relatively large amount of bone had formed after the last LAG. This new bone in the OCL, corresponds to around 50% of the bone deposited between the two previous LAGs (Fig. 2b). Considering that LAGs are formed annually, we can estimate that ovulation happens approximately six months after the last LAG, *i.e*. about six months after the last austral summer, at the beginning of August (Fig. 6.5). We hypothesize that after egg laying and hatching (Fig. 6.6), the chicks grew rapidly to reach a relatively large size so that they were better able to withstand the environmental stress of the next austral summer (Fig. 6.7), which usually corresponds to the cyclone period, and hence resource limitations.

**Figure 6:**
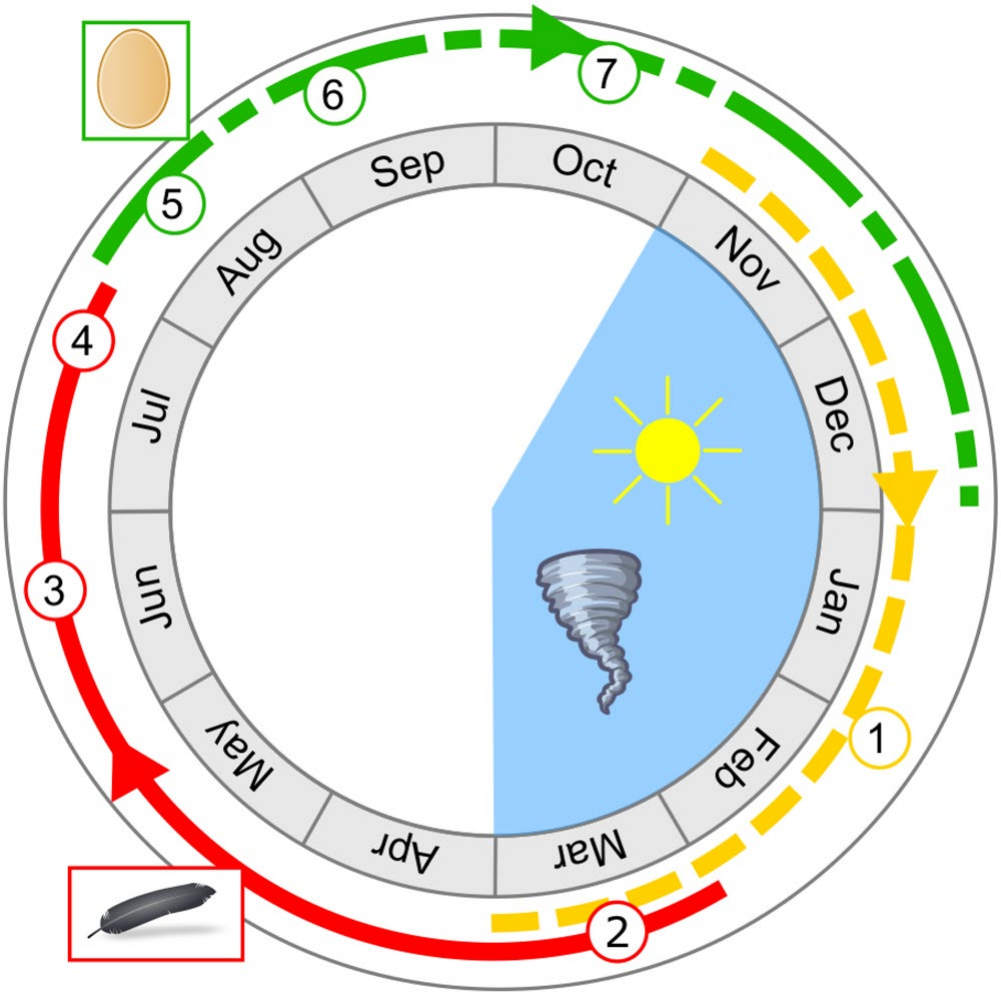
Diagram summarizing the main conclusions of this research. Blue area: indicates the summer period when storms occur between November and March. Yellow line (----): indicates when the LAGs could be formed due to the storms during the Austral Summer; Red line (−): indicates the possible molting period, after summer; Green line (-.-.-): indicates the probable breeding period. 1 – LAG formation; 2 – Beginning of molting; 3 – Mix between downy plumage and new feathers as described in the historical documents; 4 – End of the molting period, when all the feathers are renewed, as described in the historical literature; 5 – In the case of females, the breeding period start with ovulation; 6 – The eggs are laid, hatching later occurs; 7 – The chicks grow rapidly before the next cyclone season.

The external cortex of specimens that exhibit evidence of molting in their bone (i.e., several large resorption cavities) have thin lamellar deposits after the last LAG in the OCL (Figs 4 and 5), which further suggests that molting starts just after the last austral summer (Fig. 6.2). Therefore, molting occurred after the breeding and cyclone season, allowing for the renewal of plumage before the next breeding period. This is congruous with the variable descriptions of the plumage of the dodo by mariners in which they described dodos with a downy body and some feathers in the wings and the tail^49^ during June and July. These descriptions likely correspond to the middle of the molting period, when all old feathers have already been lost and replaced by temporary downy plumage (Fig. 6.3). The new feathers then appear, starting with the wings and the tail, as seen in modern birds^3^. Mariners further described a plumage composed of real feathers in the documents written in July and September^49^. This means that at the end of July the molt is completed and the breeding season begins with a new plumage (Fig. 6.4). On the basis of this evidence we propose that the dodo molt occurred between March and the end of July, after the austral summer and cyclone period, and before the next breeding season, which is consistent with the breeding and molting periods described for all surviving birds on Mauritius^3, 33, 34, 41^.

## Conclusion

This study of the bone histology of the dodo provides insight into the life history of this recently extinct bird. In order to deduce the timing of the events such as reproduction and molting we have considered the histological patterns, modern birds in Mauritius and the ecology of the area. From these we propose that the breeding season started several months before the austral summer (around August) with ovulation in the females, and that it occurred after a period of potential fattening, which corresponds with the fat and thin cycles recorded in many Mauritian vertebrates, both living and extinct. We further suggest, that after the eggs were laid and chicks hatched, they grew quickly to almost adult body size and attained sexual maturity before the cyclone period in the austral summer. Additionally, our findings could indicate that following the breeding season and the end of the austral summer, molting began (around March) with the replacement of the feathers of the wings and the tail first. Thus, at the end of July, the molt would have been completed in time for the next breeding season. These novel findings about the life history of the dodo have been deduced from the bone microstructure and the proposed timing thereof appear to correlate well with the current observations of modern birds in Mauritius, and have been further corroborated by historical descriptions.

## Materials and Methods

### Materials

Twenty-two sections from the diaphysis of dodo bones were sampled (Supplementary Table S1). The material corresponds to 22 bones, including five femora, fourteen tibiotarsi, two tarsometatarsi, and one humerus. These bones are from different fossil localities on Mauritius. The brown specimens are from the swamp named Mare aux Songes (MAS) in the south-east of the island. Some of these are held at Omnicane, Mauritius (formerly Mon Trésor Mon Désert sugar estate) and were loaned to NHM (Natural History Museum, London) for thin sectioning. Some were loaned from the Muséum d’Elbeuf (Normandie, France). The latter were discovered by the Mauritian naturalist, Paul Carié, owner of the Mare aux Songes during the 19^th^ century, and remained unknown until 2014, when the descendants of Carié donated them to the Muséum d’Elbeuf. The four white tibiotarsi are from different caves in the island. Although these specimens are fragmentary and unassociated, comparison with the Strickland and Melville^5^ anatomical descriptions of the dodo permitted their unambiguous identification.

### Methods

All the bones were sectioned in transverse section with a handheld Dremel sectioning tool in the middle of the diaphysis. For specimen “ddhu1”, in which the middle part of the diaphysis was missing, the section was taken in the most proximal part. The sectioned bits of bone were pretreated by soaking in six, eight hour baths of ethanol 95%, followed by three baths of acetone 95%, to remove the organic remains. The bone samples were thereafter embedded in Struers epoxy resin and then thin sections were prepared using an Imptech PC10 thin sectioning machine, and the final polishing was completed using silicon dioxide power on a velvet cloth^13, 51^. The thin sections were examined under a Nikon Eclipse Biological petrographic microscope E200. Microphotographs and measurements were performed using NIS Elements version 3.0 and Bone Profiler version 4.5.8.

## Electronic supplementary material

Supplementary information
