# Supplementary material for: Bone histology sheds new light on the ecology of the dodo (Raphus cucullatus, Aves, Columbiformes)
Source: Sci Rep. 2017 Aug 24;7:7993. doi: 10.1038/s41598-017-08536-3 (PMC5570941; doi:10.1038/s41598-017-08536-3)
Supplement: Supplementary file 1 — Supplementary information [file 41598_2017_8536_MOESM1_ESM.pdf]

Supplementary Tables

Title: Bone histology sheds new light on the ecology of the dodo (*Raphus cucullatus*, Aves, Columbiformes)

Authors: D. Angst[1*], A. Chinsamy[1], L. Steel[2] & J. P. Hume[3]

Affiliations:

[1] Department of Biological Sciences, University of Cape Town, Private Bag X3, Rhodes Gift, 7701 South Africa.

[2] Department of Earth Sciences, Natural History Museum, Cromwell Road, London SW7 5BD.

[3] Bird Group, Department of Life Sciences, Natural History Museum, Tring, Herts HP23 6AP.

*angst.delphine@gmail.com

Supplementary Table S1: General information on the dodo bones studied.

MAS: Mare aux Songes, Omnicane: Mon Desert Mon Tresor Sugar Estate, - (hyphen) denotes poor histological preservation

Sample number	Collection number	Bone	Collections	Origin	Maturity
ddfem01	-	femora	Muséum d'Elbeuf	MAS	adult
ddfem02	-	femora	Muséum d'Elbeuf	MAS	adult
ddfem03	-	femora	Omnicane	Cave	adult
ddfem04	MAS 39590 CH6-2	femora	Omnicane	MAS	adult
ddfem05	MAS1 - P14964	femora	Omnicane	MAS	adult
ddhu01	-	humerus	Omnicane	MAS	adult
ddtbt01	-	tibiotarsus	Muséum d'Elbeuf	MAS	adult
ddtbt02	-	tibiotarsus	Muséum d'Elbeuf	MAS	adult
ddtbt03	-	tibiotarsus	Omnicane	MAS	adult
ddtbt04	-	tibiotarsus	Omnicane	MAS	adult
ddtbt05	-	tibiotarsus	Omnicane	MAS	adult
ddtbt06	-	tibiotarsus	Omnicane	MAS	adult
ddtbt07	-	tibiotarsus	Omnicane	MAS	-
ddtbt08	-	tibiotarsus	Omnicane	MAS	adult
ddtbt09	-	tibiotarsus	Omnicane	MAS	adult
ddtbt10	FLMR R2071	tibiotarsus	Omnicane	Cave	adult
ddtbt11	FLMR R2071	tibiotarsus	Omnicane	Cave	-
ddtbt12	FLMR R2072	tibiotarsus	Omnicane	Cave	-
ddtbt13	MAS 39589 CH6-1	tibiotarsus	Omnicane	MAS	adult
ddtbt14	MAS2 - P14965	tibiotarsus	Omnicane	MAS	juvenile
ddtmt01	-	tarsometatarsus	Muséum d'Elbeuf	MAS	adult
ddtmt02	-	tarsometatarsus	Muséum d'Elbeuf	MAS	adult

Supplementary Table S2: Measurements of the bones.

ICL: Inner Circumferential Layer, OCL: Outer Circumferential Layer, LAG: lines of arrested of growth, “-”: used when the value is not observable because the thin section was badly preserved or because of significant secondary reconstruction.

Sample number	Bone circumference (mm)	Largest diameter (mm)	Smallest diameter (mm)	Cortex thickness (mm)	SD cortex thickness (µm)	Proportion of ICL (%)	Proportion of OCL (%)	Number of LAG(s)
ddfem01	55.2	9.1	8.2	1.9	0.27	30	12	2
ddfem02	51.8	8.8	8.0	2.2	0.32	28	8	1
ddfem03	49.1	8.6	7.1	2.8	0.62	-	-	1
ddfem04	56.6	9.2	8.9	2.5	0.24	31	5	1
ddfem05	52.3	8.2	7.7	1.9	0.30	35	20	1
ddhu01	41.8	4.2	3.4	0.8	0.17	28	8	1
ddtbt01	51.7	8.2	8.0	2.6	0.29	23	7	4
ddtbt02	45.8	7.9	7.0	2.4	0.36	-	24	5-6
ddtbt03	45.3	7.6	7.2	1.8	0.24	17	29	3
ddtbt04	42.8	7.6	6.8	2.2	0.37	15	12	1
ddtbt05	49.4	8.3	7.6	2.7	0.17	51	18	2
ddtbt06	50.6	8.3	8.1	2.3	0.29	21	16	2
ddtbt07	-	7.3	6.8	2.0	0.19	-	-	-
ddtbt08	48.3	8.1	7.5	2.0	0.27	15	22	2
ddtbt09	46.5	7.9	7.2	2.2	0.20	-	-	2
ddtbt10	46.5	7.7	7.1	2.3	0.34	28	16	4
ddtbt11	-	7.5	6.8	1.8	0.20	-	-	-
ddtbt12	45.5	7.4	7.0	2.2	0.33	-	-	-
ddtbt13	46.5	7.9	7.5	3.1	0.44	14	11	2
ddtbt14	48.0	7.7	7.5	2.6	0.53	31	-	-
ddtmt01	39.2	7.4	5.3	2.0	0.27	26	-	-
ddtmt02	38.1	7.2	5.0	1.6	0.22	27	9	-

Supplementary Table S3: Resorption cavities and medullary bone measurements.

MB: medullary bone, RC: resorption cavity, -: used when the value is not observable because the thin section was badly preserved or because of extensive secondary reconstruction. The proportions of resorption cavities or of medullary bone present in the thin sections were calculated as follows: proportion RC (%) = [sum RC area (mm^2)/cortex area (mm^2)] *100 and proportion MB (%) = [MB area (mm^2)/ cortex area (mm^2)] *100

Sample number	Number RC	Min RC area (mm^2)	Max RC area (mm^2)	Sum RC area (mm^2)	Proportion RC in the bone (%)	Proportion MC in the bone (%)
ddfem01	-	-	-	-	-	-
ddfem02	-	-	-	-	-	-
ddfem03	57	0.01	0.86	6.80	5.92	-
ddfem04	-	-	-	-	-	1.46
ddfem05	-	-	-	-	-	-
ddhu01	-	-	-	-	-	-
ddtbt01	-	-	-	-	-	-
ddtbt02	8	0.01	0.98	2.53	3.26	-
ddtbt03	-	-	-	-	-	-
ddtbt04	-	-	-	-	-	-
ddtbt05	2	0.09	0.09	0.18	0.15	-
ddtbt06	5	0.01	0.36	0.69	0.69	-
ddtbt07	-	-	-	-	-	-
ddtbt08	-	-	-	-	-	4.50
ddtbt09	8	0.01	0.75	1.30	1.46	-
ddtbt10	-	-	-	-	-	-
ddtbt11	-	-	-	-	-	-
ddtbt12	-	-	-	-	-	-
ddtbt13	-	-	-	-	-	-
ddtbt14	-	-	-	-	-	-
ddtmt01	-	-	-	-	-	-
ddtmt02	-	-	-	-	-	-
